# The Role of c-MYC in B-Cell Lymphomas: Diagnostic and Molecular Aspects

**DOI:** 10.3390/genes8040116

**Published:** 2017-04-05

**Authors:** Lynh Nguyen, Peter Papenhausen, Haipeng Shao

**Affiliations:** 1 Department of Hematopathology and Laboratory Medicine, H. Lee Moffitt Cancer Center and Research Institute, Tampa, FL 33612, USA; lynh.nguyen@moffitt.org; 2 Cytogenetics Laboratory, Laboratory Corporation of America, Research Triangle Park, NC 27709, USA; papenhp@labcorp.com

**Keywords:** c-MYC, lymphoma, diffuse large B-cell lymphoma, plasmablastic lymphoma, mantle cell lymphoma, double-hit lymphoma, complex karyotype

## Abstract

c-MYC is one of the most essential transcriptional factors, regulating a diverse array of cellular functions, including proliferation, growth, and apoptosis. Dysregulation of *c-MYC* is essential in the pathogenesis of a number of B-cell lymphomas, but is rarely reported in T-cell lymphomas. *c-MYC* dysregulation induces lymphomagenesis by loss of the tight control of *c-MYC* expression, leading to overexpression of intact c-MYC protein, in contrast to the somatic mutations or fusion proteins seen in many other oncogenes. Dysregulation of *c-MYC* in B-cell lymphomas occurs either as a primary event in Burkitt lymphoma, or secondarily in aggressive lymphomas such as diffuse large B-cell lymphoma, plasmablastic lymphoma, mantle cell lymphoma, or double-hit lymphoma. Secondary *c-MYC* changes include gene translocation and gene amplification, occurring against a background of complex karyotype, and most often confer aggressive clinical behavior, as evidenced in the double-hit lymphomas. In low-grade B-cell lymphomas, acquisition of *c-MYC* rearrangement usually results in transformation into highly aggressive lymphomas, with some exceptions. In this review, we discuss the role that c-MYC plays in the pathogenesis of B-cell lymphomas, the molecular alterations that lead to *c-MYC* dysregulation, and their effect on prognosis and diagnosis in specific types of B-cell lymphoma.

## 1. Introduction

The proto-oncogene *c-MYC*, located at chromosome 8q24, is one of the genes most frequently involved in human carcinogenesis. The *c-MYC* gene was initially identified as the cellular homolog of the *v-myc* oncogene in avian acute leukemia virus (MC29) in 1978 [1,2]. Direct evidence of *c-MYC*’s involvement in human cancer came from the discovery and identification of the *c-MYC* gene at 8q24 and its translocation onto the immunoglobulin heavy chain locus in human Burkitt lymphoma [3,4,5]. Subsequent studies demonstrated that the *c-MYC* gene, coupled with the immunoglobulin μ or κ enhancer in transgenic mice, was highly leukemogenic and resulted in the development of fatal B-cell lymphomas [6]. Over the past three decades, c-MYC has been shown to be an essential global transcription factor regulating 10–15% of all human genes [7]. c-MYC controls a variety of cellular functions, including cell cycle, cell growth, survival, cellular metabolism and biosynthesis, adhesion, and mitochondrial function [8].

Due to its central role in human cells, c-MYC is tightly regulated at both the transcriptional and translational levels [9]. The *c-MYC* gene has three exons: exon 1 is non-coding and has two promoters; exons 2 and 3 encode the c-MYC protein with translation initiation at nucleotide 16 of exon 2. There are four *c-MYC* transcriptional promoters with promoter P2 contributing to approximately 80–90% of total *c-MYC* RNA in normal cells [10]. Both *c-MYC* messenger RNA (mRNA) and c-MYC protein have very short half-lives in normal cells [11,12,13]. Without appropriate positive regulatory signals, c-MYC protein levels are low and insufficient to promote cellular proliferation. The transforming activity of c-MYC is also counteracted by its ability to induce apoptosis under normal physiological conditions [14]. In c-MYC-induced cancers, this delicate balance of c-MYC regulation is lost. However, unlike other proto-oncogenes, c-MYC is not activated by oncogenic mutations in the coding sequence. c-MYC transforms cells via unregulated overexpression of intact c-MYC protein through three main mechanisms: insertional mutagenesis, gene amplification, and chromosomal translocation. Insertional mutagenesis is seen in retrovirus-induced tumors, such as avian leucosis virus (ALV)-induced hematopoietic tumors, in which the proviral enhancer is integrated upstream of the *c-MYC* gene and leads to c-MYC overexpression [15]. Amplification of *c-MYC* gene has been shown in both hematopoietic and non-hematopoietic tumors, including lung, breast, and colon cancers [16,17,18,19,20,21]. Chromosomal translocations juxtaposing the *c-MYC* gene locus at chromosome 8q24 with immunoglobulin genes at chromosome 14q32, 2p11, and 22q11 or other partner genes are by far the most common and well-studied. The translocations result in deregulated expression of c-MYC [22].

c-MYC regulates downstream gene expression in a tissue specific manner with little overlap in genes in different cell types [23]. This can be explained by findings that indicate c-MYC functions as a universal amplifier of already expressed genes in cells rather than directly activating silent genes [24,25]. In hematopoietic malignancies, genomic abnormalities involving the *c-MYC* gene are almost always seen in B-cell lymphomas. In contrast, *c-MYC* genetic alterations are rarely reported in T-cell lymphomas. This review summarizes the role of c-MYC in B-cell lymphomas and leukemias, particularly in relation to the specific subtypes classified under the 2016 revision of the World Health Organization (WHO) classification of lymphoid neoplasms [26].

## 2. c-MYC in B-Cell Development

B-cells are derived from hematopoietic stem cells in the bone marrow. Early B-cells in the bone marrow undergo antigen independent progressive development characterized by immunoglobulin gene rearrangement and expression of stage specific surface markers. The mature naïve B-cells exit the bone marrow and upon encountering antigens in lymphoid tissue develop into germinal center B-cells. Germinal centers (GC) are sites of B-cell proliferation and selection for memory B-cells and plasma cells with high affinity receptor/antibodies in a T-cell antigen-dependent manner [27]. The naïve B-cells are first stimulated by antigen and antigen presenting helper cells to transform into centroblasts in the “dark zone” of GC [27]. The centroblasts undergo rapid cellular division and progressively modify their surface antigen receptors through somatic hypermutation of immunoglobulin genes (specifically the variable region (IgV)), and subsequently give rise to non-proliferating centrocytes in the “light zone” of GC. The B-cell development in the GC is tightly regulated by key transcription factors, such as B-cell lymphoma 6 protein (BCL6) and multiple myeloma 1/interferon regulatory factor 4 protein (IRF4/MUM1). BCL6 is expressed in GC B-cells and is necessary for GC formation [28,29]. IRF4/MUM1 is required for class-switch recombination and differentiation of GC B-cells into plasma cells [30]. BCL6 and IRF4/MUM1 are sequentially activated and mutually exclusive in normal GC B-cells [31]. In the GC, the somatic hypermutation and class-switch recombination of the immunoglobulin genes are prone to oncogenic genetic changes leading to the development of B-cell lymphoma. This is probably the reason that the majority of B-cell lymphomas are derived from GC or post GC B-cells.

c-MYC is essential for early B-cell development in the bone marrow via activation of a transcription factors essential for the maintenance of B-cell identity, early B-cell factor 1 (*ebf-1*) [32]. However, the role of c-MYC in GC development is unclear as c-MYC expression is not detected in highly proliferating GC B-cells, which contradicts the central role of c-MYC in cellular proliferation [33]. Recent studies shed light on the role of c-MYC in GC formation and maintenance. Dominguez-Sola and Calado et al. confirmed the absence of c-MYC expression in the centroblasts located in the GC “dark zone”, but showed c-MYC expression in a small subset of GC light zone B-cells [34,35]. Both groups showed that GC B-cells with c-MYC expression were required to maintain the GC. According to these studies, in the early stage of GC development c-MYC is temporarily expressed in a small subset of B-cells through interactions with antigens and T-helper cells. Transient activation of surface receptors such as B-cell receptor (BCR), cluster designation 40 (CD40), and interleukin (IL)-2 receptors that initiate GC reaction suppresses BCL6 activity, which ultimately alleviates the negative regulation of *c-MYC* by BCL6, and allows the GC B-cells to undergo the first round of cell division [36,37]. In c-MYC^+^ cells, the nuclear factor (NF)-κB pathway is activated, which leads to transcriptional activation of *c-MYC* and *IRF4*, providing a positive feedback for c-MYC expression. At this stage, BCL6 and MYC appear to be co-expressed in the light zone GC B-cells. In these cells, c-MYC activates both cyclin D2 (*CCND2)* in addition to cyclin D3 (*CCND3*), resulting in a *CCND2*-dependent proliferation. The T-helper cells induce the c-MYC^+^ GC B-cells in the light zone to re-enter the dark zone for additional rounds of positive selection by undergoing proliferation and somatic hypermutation. c-MYC expression is required for this re-entry to sustain the GC reaction. *BCL6* is subsequently upregulated to initiate the GC formation, and BCL6 suppresses c-*MYC* transcription directly by binding to its promoter [38,39,40].

In the GC dark zone, c-MYC is completely suppressed in proliferating GC centroblasts. The transcription factor E2A immunoglobulin enhancer-binding factors (TCF3) is expressed in the GC dark zone B-cells and activates *CCND3* and *E2F2*, which replace the CCND2-dependent proliferation in *c-MYC*+ GC B-cells [37]. BCL6 also induces a MYC-independent cell cycle progression by binding with transcription factor MYC-interacting zinc finger protein 1 (MIZ1), which is a MYC partner to suppress cyclin-dependent kinase (CDK) inhibitors [41,42]. c-MYC must be actively repressed in GC dark zone to limit the numbers of cell division before each round of antigen affinity-based selection [43], and allow normal affinity maturation to proceed, as c-MYC negatively regulates the transcriptional pause release of RNA polymerase II, which is essential for AID-mediated somatic hypermutation [44]. TCF3 induces the expression of inhibitor of DNA binding 3 protein (ID3), which is an inhibitor of TCF3. ID3 promotes the migration of GC B-cells from the dark zone to the light zone. The c-MYC^−^ cells in the light zone exit the GC and become memory B-cells or plasmablasts. B-lymphocyte-induced maturation protein 1 (BLIMP1) activation in post GC early plasmablasts suppresses c-MYC expression and induces plasma cell differentiation. Abnormalities in AID activity at actively transcribed *c-MYC* gene locus can lead to translocations that juxtapose the Ig enhancer with the 5’ region of *c-MYC*, thereby abolishing the BCL6 mediated suppression of c-MYC expression in the GC dark zone [35]. The unregulated expression of c-MYC in GC B-cells leads to the bypass of affinity-based selection and perpetuation of the GC re-entry that significantly increases the chances of oncogenic genetic events for the development of B-cell lymphoma.

## 3. Clinical Detection of c-MYC Abnormalities

The detection of *c-MYC* gene translocation and c-MYC protein expression has become essential in the clinical diagnosis and prognosis of aggressive B-cell lymphomas [26]. Two techniques are clinically available for the detection of *c-MYC* gene abnormalities: conventional karyotyping and fluorescence in situ hybridization (FISH). Karyotyping is valuable in assessing the gross genomic changes of lymphoma cells, such as ploidy, deletions, translocations and marker chromosomes. The advantage of using karyotyping is its ability to determine the partner chromosome of the *c-MYC* translocation. The partner gene of *c-MYC* translocation can sometimes be inferred from the segment of the partner chromosome involved in the translocation and later confirmed by FISH if appropriate probes are available. The complexity of the genomic changes is also useful in differentiating Burkitt lymphoma from high-grade B-cell lymphoma, not otherwise specified (NOS). A complex karyotype with *c-MYC* rearrangement, especially with chromosomal partners other than one of the immunoglobulin gene loci (chromosomes 14q, 2p and 22q) can indicate genomic evolution of the lymphoma cells into a high-grade B-cell lymphoma, NOS rather than development of Burkitt lymphoma [45]. The main disadvantage of conventional karyotyping study is that it requires fresh tissue, which is infrequently available as tissue is routinely fixed in formalin right after biopsy or surgery in daily clinical practice. For this reason, FISH is the most commonly used method in detecting *c-MYC* gene abnormalities. FISH can be performed on suspended viable cells or formalin-fixed paraffin-embedded (FFPE) tissue sections from routine tissue blocks. FISH is highly sensitive and has a faster turnaround time, usually within one day. FISH detects *c-MYC* gene translocation with either a dual-color break-apart probe set or a dual fusion probe set. The *c-MYC* dual fusion probe set targets *c-MYC* and immunoglobulin heave chain IGH genes [t(8;14)(q24;q32)] specifically, and is usually preferred for confirmation of histologic diagnosis of Burkitt lymphoma [46]. A dual-color break-apart *c-MYC* probe set can detect most *c-MYC* breaks, but cannot identify the partner gene(s). High-throughput genetic analysis such as comparative genomic hybridization (CGH) and single nucleotide polymorphism (SNP) arrays are also available and can be performed on FFPE tissue, giving additional valuable information on *c-MYC* amplification and its copy number variation.

Despite the initial production of antibodies against c-MYC protein by Evan et al. in 1985 and the successful application in molecular biology studies [47], these antibodies against the C-terminal portions of c-MYC cannot be used for the detection of c-MYC by routine immunohistochemical stain on FFPE tissue. It appears that epitopes in the C-terminus of c-MYC protein are usually lost upon FFPE treatment [48]. Two antibodies (N262 from Santa Cruz Biotechnology (Dallas, TX, USA) and Mab Y69 from Epitomics (Cambridge, MA, USA) against the N-terminus of c-MYC can be used for immunohistochemical stain in FFPE tissue and have been shown to be sensitive and specific [48]. As expected, the c-MYC specific antibodies stain cells in a nuclear pattern. Performing immunohistochemical stain for c-MYC expression is important for prognosis in diffuse large B-cell lymphoma (DLBCL), as DLBCLs with expression of both c-MYC and B-cell lymphoma 2 protein (BCL2) (so-called “double-expressor” lymphoma) have worse clinical outcome than other DLBCL, NOS treated with rituximab, cyclophosphamide, doxorubicin, vincristine, and prednisone (R-CHOP) [49,50]. The expression of c-MYC and BCL2 in the “double-expressor” lymphomas is independent of *c-MYC* or *BCL2* gene rearrangement [51,52], and is only used as a prognostic marker rather than diagnostic criteria. c-MYC expression is considered positive when >40% of the lymphoma cells are stained [49].

c-MYC expression detected by immunohistochemistry (IHC) does not equate to *c-MYC* rearrangement. However, there is a correlation between c-MYC expression by IHC and *c-MYC* gene abnormalities in aggressive B-cell lymphomas [53]. Burkitt lymphoma as well as DLBCL with *c-MYC* rearrangement usually show diffuse, strong nuclear or nuclear/cytoplasmic immunohistochemical staining for c-MYC in the majority of cells, while DLBCLs without *c-MYC* rearrangement typically only show a subset of positive cells (<40% lymphoma cells) or nonspecific cytoplasmic staining [54,55]. Kluk et al. showed that all cases of DLBCL with c-MYC rearrangement can be identified by immunohistochemistry with a cutoff value of ≥50% c-MYC+ lymphoma cells in a series of 77 cases [56]. Green et al. proposed an optimal cutoff of ≥70% c-MYC^+^ lymphoma cells demonstrating 100% sensitivity and 93% specificity in predicting *c-MYC* rearrangement [57], while another recent study showed that immunohistochemistry was neither specific nor adequately sensitive as a surrogate for *c-MYC* rearrangement with any cutoff value [52]. There is no statistically significant difference in the percentage of c-MYC positive lymphoma cells between DLBCLs with multiple copies of *c-MYC* and those with no *c-MYC* abnormalities [52].

## 4. c-MYC in Burkitt Lymphoma

Burkitt lymphoma (BL) is a mature B-cell lymphoma with extremely high proliferation rate. In contrast to B-lymphomas with predominant lymph node involvement, BL most commonly involves extranodal sites such as the jaw, bones, gastrointestinal tract, gonads, or breasts. Histologically, BL is composed of a monotonous population of medium-sized lymphoma cells with cohesive diffuse growth interspersed with many tingible body macrophages in a characteristic “starry sky” pattern. The lymphoma cells have slightly squared off nuclear membrane, multiple medium-sized basophilic paracentric nucleoli and deep basophilic cytoplasm. On cytological preparations, the lymphoma cells are blastoid with many cytoplasmic vacuoles. The lymphoma cells show characteristic high proliferation with abundant mitotic figures and apoptotic bodies. BL is derived from germinal center or post germinal center B-cells. The BL cells show expression of germinal center B-cell markers, cluster designation 10 CD10, BCL6, and Lin11/Isl1/Mec3 domain only 2 LMO2, and are typically negative for BCL2. The BL cells show extremely high proliferation rate by Ki-67 immunostain (>95% positive cells). There are three clinical variants of BL: endemic (African-derived), sporadic, and human immunodeficiency virus (HIV)-associated BL differing in geographic location, preferential sites of involvement, and frequency of Epstein–Barr virus infection [58].

*c-MYC* rearrangement with one of the immunoglobulin gene loci is the genetic hallmark of BL. The *c-MYC* rearrangement in BL is most often associated with simple karyotype [59]. In BL, the translocation of *c-MYC* onto the immunoglobulin heavy chain gene locus at 14q32 is the most common, occurring in approximately 80% cases, while variant translocations of *c-MYC* with the immunoglobulin kappa or lambda light chain genes at 2p12 or 22q11, respectively, occur in approximately 10% of cases. In BL, there are three main translocation breakpoints in *c-MYC* [60]. The class I breakpoints are within the exon 1 and first intron of *c-MYC*; the class II breakpoints are located at the 5′ end of the *c-MYC*, and usually within a few kilobases of exon 1; and the class III breakpoints are distant from *c-MYC* itself, and can be more than 100 kilobases away. The clinical variants of BL demonstrate different preferential translocation breakpoints in both *c-MYC* and the partner immunoglobulin genes [61,62]. Endemic BL typically shows class II translocation breakpoints in *c-MYC*, and breakpoints at the VDJ region of immunoglobulin gene. The sporadic variant of BL often exhibits class I breakpoints of *c-MYC* and breakpoints at the class switch region of immunoglobulin gene. In endemic BL, the intron enhancer of the immunoglobulin heavy chain gene is on the same chromosome as the translocated *c-MYC* and contributes to the unregulated c-MYC expression. In sporadic BL, the IGH enhancer intron is on the opposite chromosome of the translocation and is not able to drive c-MYC expression; instead, the enhancers at 3′ end of the immunoglobulin heavy chain and kappa light chain genes are always located on the same chromosome as *c-MYC* and likely lead to *c-MYC* deregulation [63]. In contrast to normal cells, there is loss of inhibition of transcriptional elongation at P2 promoter in BL and a shift of principal transcriptional promoter from P2 to P1 [64]. The translocated *c-MYC* is located in the hypermutable immunoglobulin locus and is therefore subjected to somatic hypermutations in the germinal centers [65]. In fact, most translocated *c-MYC* genes contain somatic mutations, usually point mutations or deletions in exon 1 or near the exon 1/intron 1 boundary [66,67,68]. Other hotspot non-conserved mutations in coding regions that occur as a result of somatic hypermutation can contribute to the oncogenic potential of c-MYC by increasing c-MYC protein stability through a number of different mechanisms [69,70,71]. At present time, the clinical significance of somatic hypermutations of *c-MYC* in BL and other B-cell lymphomas is not established.

A molecular Burkitt lymphoma (mBL) signature was identified by gene expression profiling studies (GEP) [72,73]. In Hummel’s study, the genes in the mBL signature were composed of 58 genes, including several target genes of the NF-κB pathway, such as B-cell lymphoma protein 2A1 (*BCL2A1*), FLICE-like inhibitory protein (*FLIP*), cluster designation 44(*CD44*), nuclear factor κBIA (*NFKBIA*), B-cell lymphoma 3 protein (*BCL3*), and signal transducer and activator of transcription 3 (*STAT3*), that all can be used to distinguish activated B-cell–like or germinal-center B-cell–like lymphomas [72]. In Dave’s study, GEP showed increased expression of *c-MYC* target genes and a subgroup of germinal-center B-cell genes in addition to low level of expression of major-histocompatibility-complex class I genes and NF-κB target genes [73]. The mBL signature did not show high concordance with histologic diagnosis of BL. The mBL included cases of classical or atypical BL as well as significant numbers of cases with morphologic features of DLBCL. In contrast, not all cases with morphological BL were classified as mBL. The mBL is clinically and biologically relevant as all the mBL cases showed *c-MYC* rearrangement with a simple karyotype background and favorable prognosis regardless of the morphology. Three cytogenetic groups can be defined by the pattern of chromosomal abnormalities in Hummel’s study [72]. The first group, “MYC-simple” is defined as lymphomas with immunoglobulin (IG)-MYC fusions and a low chromosomal complexity score (<6) that lacked *IGH*-*BCL2* fusions and *BCL6* breakpoints. The second group, “MYC-complex” included lymphomas with IG-MYC fusions or non–*IG*-*MYC* fusions that have a high chromosomal complexity score (≥6), an *IGH*-*BCL2* fusion, *BCL6* breakpoint, or any combination of these. The third group, “MYC-negative,” included MYC-negative lymphomas. The mBL cases consisted mostly of “MYC-simple” lymphomas, while the non-mBL cases were predominantly “MYC-negative” lymphomas. The intermediate group as defined by mBL signature comprised most of the “MYC-complex” lymphomas. The mBL or “MYC-simple” showed significantly improved five-year survival rate compared to the non-mBL or intermediate lymphomas.

The mBL signature confirmed the presence of rare cases of histologically BL lacking *c-MYC* rearrangement, but instead had chromosome 11q aberrations characterized by interstitial gains at 11q23.2-q23.3 and telomeric losses of 11q24.1-qter [74,75]. Gains at 11q23 are associated with overexpression of genes including platelet-activating factor acetylhydrolase IB subunit beta (*PAFAH1B2)*, while telomeric losses contained a focal homozygous deletion in 11q24.2-q24.3 including the ETS proto-oncogene 1 (*ETS1)* gene. These cases shared similar clinical course with typical BL, but more often showed nodal disease and complex karyotype. Due to the limited cases reported to date, additional studies are needed to better understand these lymphomas. Currently, these cases are classified as Burkitt-like lymphoma with 11q aberration under the revised 2016 WHO classification [26].

Next generation sequencing studies have identified mutations in *TCF3* and *ID3* in approximately 70% of sporadic and HIV-related BL and 40% of endemic BL [76,77,78]. ID3 inhibits the transcriptional activity of TCF3. TCF3, as a master regulator of germinal center B-cell differentiation, is constitutively activated by somatic mutations in *TCF3,* blocking ID3 binding, and also by inactivating mutations in *ID3*. Constitutive activation of *TCF3* promotes tonic BCR signaling by repressing protein tyrosine phosphatase, non-receptor type 6 (*PTPN6*), which encodes a negative regulator of BCR signaling, phosphatase SHP-1. The tonic activation of BCR signaling induces phosphatidylinositol 3-kinase signaling pathways to promote survival of BL cells. TCF3 also activates CCND3 to drive BL cells through cell cycle progression. Somatic mutations in *CCND3* also play a role in BL lymphomagenesis as these mutations are present in approximately 30% of BL and function to stabilize CCND3 expression [79].

c-MYC overexpression suppresses antigen presentation through the human leukocyte antigen (HLA) class II pathway in BL and likely other B-cell lymphomas [80]. c-MYC overexpression results in alteration of the intracellular components of the class II pathway, including decreased levels of class II editor human leukocyte antigen DM (HLA-DM), lysosomal thiol-reductase GILT, and a 47-kDa enolase-like protein. This leads to disruption of antigen/peptide presentation to CD4^+^ T-helper cells and decreased class II-mediated immunogenicity in BL, which contributes to the immunoevasive properties of BL cells.

## 5. c-MYC in Diffuse Large B-Cell Lymphoma and Double-Hit Lymphoma

**Figure 1:**
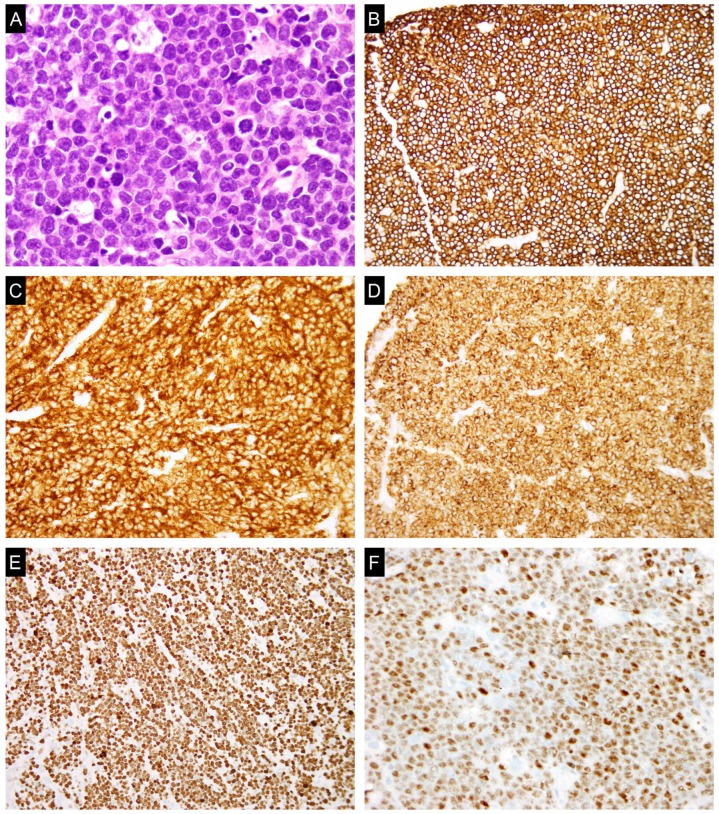
A typical case of double-hit lymphoma (DHL). (**A**) Hematoxylin and eosin (H&E) section shows sheets of medium-sized lymphoid cells with blastoid chromatin and mitotic figures. The lymphoma cells are positive for B-cell marker cluster differentiation 20 (CD20) (**B**); germinal center marker (cluster differentiation 10 (CD10) (**C**); B-cell lymphoma protein 2 (BCL2) (**D**); and c-MYC (**F**); and show a high proliferation rate by Ki-67 (>90%) (**E**).

**Figure 2:**
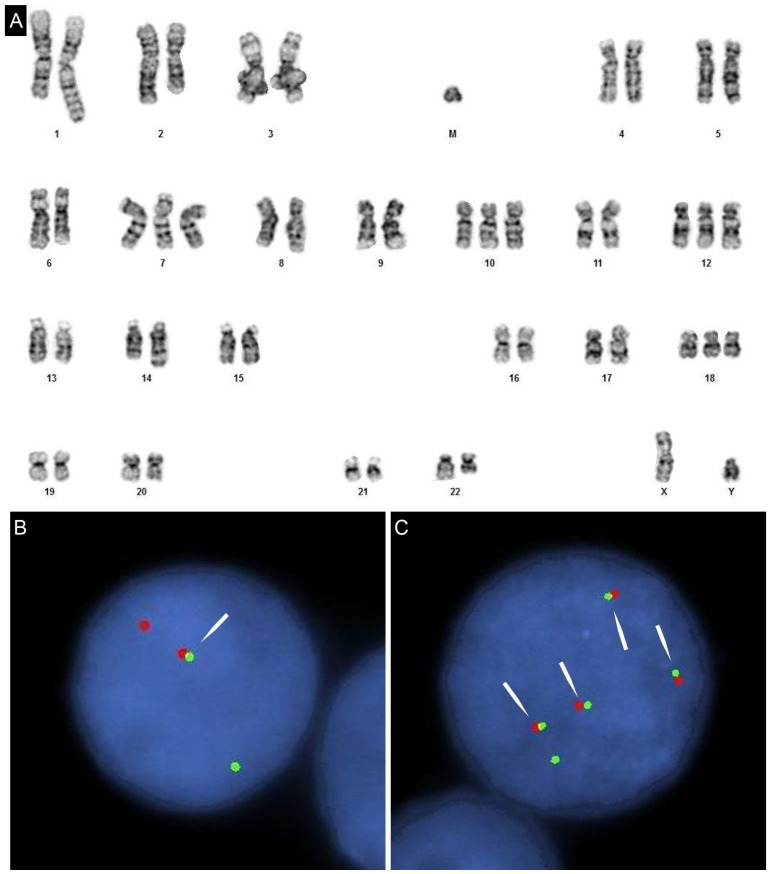
(**A**) Karyotype of DHL with *c-MYC*/*IGL* and *BCL2*/*IGH* rearrangements. Clonal evolution related triplication of the derivative (18) t(14;18) highlights the complex additional alterations; (**B**) fluorescent in-situ hybridization (FISH) targeting *c-MYC* using a flanking break-apart probe set. Fusion (arrow) represents the normal locus. The separated red and green signals indicate *c-MYC* rearrangement; (**C**) Dual fusion FISH targeting immunoglobin heave chain (*IGH*) (green) and *BCL2* (red). One fusion signal represents derivative 14 and the remaining three fusions signals are the oncogenic 18 derivatives (arrows). Absence of a normal 18 (an isolated red signal) agrees with the karyotype and is likely due to mitotic recombination-based evolution, transferring the fusion site to the original normal homolog, in addition to the more common nondisjunction-based duplication for the third derivative in this etiology.

DLBCL, NOS is defined as a mature B-cell lymphoma with diffuse growth pattern composed of large B lymphoid cells. It accounts for approximately 25–30% of non-Hodgkin lymphoma in Western countries. DLBCL, NOS is a biologically heterogeneous group of lymphomas. Two major groups of DLBCL, NOS are identified by GEP: germinal center B-cell like (GCB-like) and activated B-cell like (ABC-like) subgroups [81]. The GCB-like DLBCLs express genes characteristic of germinal center B-cells and have more favorable prognosis. ABC-like DLBCLs express genes in activated peripheral B-cells and have adverse prognosis. Because GEP is not available for routine clinical use, several algorithms by immunohistochemistry with antibodies against germinal center and post germinal center antigens were developed to replicate the sub-classification of DLBCL by GEP [82,83]. The Hans algorithm with CD10, BCL6 and MUM1 immunohistochemistry is the most commonly used in clinical practice and has a concordance rate of approximately 80% with GEP [82]. The International Prognostic Index (IPI) and revised IPI (R-IPI) are based on patient age, lactate dehydrogenase (LDH), number of extranodal sites, Ann Arbor stage, and Eastern Cooperative Oncology Group (ECOG) performance status and remain the main clinical tools in predicting prognosis in patients treated with CHOP chemotherapy (cyclophosphamide, doxorubicin, vincristine, and prednisone) [84,85].

t(14;18)(q32;q21) with *BCL2* rearrangement to *IGH* gene locus is present in approximately 20–30% of cases of DLBCL [86,87,88]. *BCL2* rearrangement is present in approximately 30% of cases of GCB-like subgroup and less than 5% of ABC-like DLBCL. *BCL6* rearrangement at chromosomal locus 3q27 is seen in approximately 30% of cases of DLBCL [89,90]. *BCL6* rearrangement is less specific for GCB-like DLBCL. *c-MYC* rearrangement is identified in 5–15% of cases of DLBCL, NOS [88,91,92,93,94,95]. c-MYC protein expression is seen in a higher percentage (approximately 30–50%) of cases of DLBCL [53,96], suggesting mechanisms other than rearrangement in the activation of c-MYC expression, such as microRNA amplification or mutations. *c-MYC* rearrangement in DLBCL is associated with inferior progression-free survival and overall survival in patients treated with R-CHOP chemotherapy [72,97,98]. DLBCLs with *c-MYC* rearrangement have an increased risk of relapse in the central nervous system independent of other risk factors [97]. Similarly, *c-MYC* amplifications, but not *c-MYC* gains are associated with unfavorable prognosis [98]. Of note, the *c-MYC* rearrangement in DLBCL is often present in the context of a complex karyotype [99,100]. The adverse prognosis associated with *c-MYC* rearrangement is largely derived from concurrent *BCL2* or *BCL6* rearrangements [101,102,103]. The large B-cell lymphomas with *c-MYC* and either *BCL2* or *BCL6* rearrangements are subsequently termed double-hit lymphomas (DHL), while those with all three rearrangements are referred to as triple-hit lymphomas (THL). DHL typically refers to both DHL and THL, and are currently classified as high-grade B-cell lymphoma, with *MYC* and *BCL2* and/or *BCL6* rearrangements in the 2016 revision of the WHO classification of lymphoid neoplasms [26]. *BCL2* rearrangement is present in approximately 80–90%, *BCL6* rearrangement in about 5% of cases of DHL/THL, and THL comprises about 8% of all DHL/THLs [104,105,106,107]. The incidence of DHL is approximately 10% in de novo DLBCL, and about 20% in high grade lymphoma transformed from low grade B-cell lymphoma, especially follicular lymphoma (FL) [102,108]. DHLs are very aggressive lymphomas with dismal clinical outcome. Patients with DHLs are refractory to most chemotherapy regimens and die within the first year of diagnosis. The poor prognosis of DHL is independent of IPI, which is usually high in these patients [109]. Histologically, most DHLs show features intermediate between DLBCL and Burkitt lymphoma. The lymphoma cells are often medium-sized with blastoid chromatin and are associated with brisk mitoses (Figure 1). The proliferation rate usually is very high. However, there are cases of DHLs with morphologic features of typical DLBCL, NOS. Therefore, histology is unreliable in identifying DHLs. FISH is both sensitive and specific and is the gold standard in diagnosing DHL. Nearly all DHLs (93%) are of germinal center origin showing expression of CD10, but DHLs with *c-MYC* and *BCL6* rearrangements are more likely to be CD10 negative, IRF4/MUM1 positive, less commonly BCL2 positive, and cytogenetically less complex [103]. As there is a correlation between high-level c-MYC expression by immunohistochemistry and *c-MYC* rearrangement, there is increased chance that GCB-like large B-cell lymphoma with high-level c-MYC expression are likely DHL. FISH should be performed in these cases to confirm the diagnosis of DHL (Figure 2). It was also advocated by some studies that FISH should be performed in all newly diagnosed DLBCL cases [110].

Gene mutational studies showed that DHLs had mutational patterns intermediate between DLBCL and BL [111]. In this study, “mutBL” pattern included mutations in BL-associated genes (*ID3/TCF3*, *CCND3* and *c-MYC*), while “mutDL” pattern included mutations in DLBCL-associated genes (*BCL2*, enhancer of Zeste homolog 2 (*EZH2*), cyclic adenosine 3,5-monophosphate response element-binding protein binding protein (*CREBBP*), E1A binding protein P300 (*EP300*), myocyte enhancer factor 2B (*MEF2B*) and serum/glucocorticoid regulated kinase 1(*SGK1*)). The majority of DHL as well as DLBCL with single hit *c-MYC* rearrangement showed combined ‘mutBL/DL’ pattern. There is a molecular difference between DHLs with *c-MYC/BCL2* rearrangement and *c-MYC/BCL6* rearrangement [112]. DHLs with *c-MYC*/*BCL2* rearrangement showed *TP53* mutation frequency intermediate between DLBCL and BL, while *TP53* mutations were scarce in DHLs with *c-MYC*/*BCL6* rearrangement. *c-MYC*/*IgH* translocation is mediated by activation-induced cytosine deaminase in germinal centers [113], and is considered to be a secondary event after *BCL2* rearrangement as suggested in cases of transformed FL with *c-MYC* rearrangement [114]. The complex karyotype commonly seen in DHL suggests accumulation of chromosomal changes involving genes such as *TP53* in addition to *c-MYC*, *BCL2* or *BCL6* are important for lymphomagenesis and prognosis [107].

High-level c-MYC protein expression is associated with inferior overall survival irrespective of IPI and BCL2 expression [56,98]. c-MYC expression shows stronger predictive value when combined with BCL2 expression [50]. The co-expression of c-MYC and BCL2 detected by immunohistochemistry is present in approximately 20-35% of cases of DLBCL [51,96]. DLBCLs with co-expression of c-MYC and BCL2 are termed “double-expressor lymphomas” and most do not have concurrent *c-MYC* and *BCL2* gene rearrangements. Double-expressor lymphomas have worse clinical outcome compared to other DLBCL, NOS in patients treated with R-CHOP [49,51]. In these studies, the cutoffs values for c-MYC and BCL2 by immunohistochemistry are set at ≥40% and ≥70% or 50%, respectively. The double-expressor lymphomas are not as aggressive as DHL. They occur more frequently in the ABC-like subgroup, which may explain the difference in prognosis of GCB-like and ABC-like DLBCL. Unregulated c-MYC expression promotes cell proliferation, but also induces apoptosis in p53-dependent and p53-independent pathways [115]. BCL2 promotes cell survival and, in combination with c-MYC expression, confers both proliferation and survival advantages in lymphoma cells, which is part of the underlying biological basis for the more aggressive clinical behavior of the double-expressor lymphomas [116].

## 6. c-MYC in Plasmablastic Lymphoma

Plasmablastic lymphoma (PBL) is a rare type of high-grade B-cell lymphoma, most commonly seen in HIV-positive patients. In this setting, PBL shows a preferential localization in the oral cavity. The clinical course is aggressive with poor prognosis. Histologically the PBL show diffuse infiltration by immunoblast-like cells with prominent central nucleoli and variable plasmacytic differentiation. Mitotic figures are brisk. In contrast to other B-cell lymphomas, PBL cells usually show no expression of B-cell markers [CD20 and paired box homeotic gene 5 (PAX5)], but instead demonstrate a plasma cell phenotype with expression of CD138, CD38, MUM1, and cytoplasmic immunoglobulins. EBV is positive in the majority of cases by EBER in situ hybridization, especially in HIV-positive cases. The proliferation rate by Ki-67 immunostain is very high, usually >90%.

During normal B-cell development, plasmablasts are derived through two different differentiation pathways: naïve B-cells with unmutated immunoglobulin M and lambda light chain (IGM) or post-germinal center B-cells with class-switched and hypermutated immunoglobulin variable heavy chain [117]. Approximately 40% of PBL show somatic IgVH hypermutation indicating progression from the germinal center, while about 60% PBL demonstrate germline *IgVH* gene consistent with derivation from naïve B-cells [117]. Of note, there is no clinical difference between these two groups of PBL. Positive regulatory domain I-binding factor 1(PRDM1)/Blimp1 is a transcriptional repressor and inducer of terminal differentiation of B-cells into immunoglobulin secreting plasma cells [118]. PRDM1/Blimp1 induces X-Box binding protein 1 (XBP1), a critical regulator of plasma cell differentiation and represses both *PAX5* to terminate B-cell programming and *c-MYC* to inhibit cell proliferation [119].

c-MYC rearrangements, most commonly t(8;14)(q24·1;q32) with *c-MYC*/*IGH* fusion, are found in approximately 49% of cases of PBL [120]. PBL shows no rearrangements of *BCL2* or *BCL6*. *c-MYC* rearrangements are more common in EBV-positive (74%) than EBV-negative (43%) PBL. There is no difference in patient survival based on the status of *c-MYC* rearrangement. Similar to DHL, *c-MYC* rearrangement is a late genetic alteration usually in the setting of complex karyotype in PBL, suggesting it is acquired during disease progression. The high proliferation rate of PBL cells can be explained by the *c-MYC* rearrangement. Similar to DLBCL, c-MYC expression in PBL is not limited to cases with *c-MYC* rearrangement or amplification. Recurrent somatic mutations in *PRDM1* are identified in approximately 50% PBL, and more frequently associated with *c-MYC* rearrangements [121]. The somatic mutations in *PRDM1* are located in functional domains regulating the target such as *c-MYC*, indicating loss of normal suppression of c-MYC expression by PRDM1 in PBL. Therefore, there appears to be cooperative relationship between genetic changes of *PRDM1* and *c-MYC* in PBL lymphomagenesis. Alterations in *c-MYC* are also frequent in plasma cell myeloma (PCM) and associated with disease progression and aggressive clinical behavior [122,123]. Taddesse-Heath et al. reported four cases with overlapping clinical findings between PBL and PCM, all with histological and phenotypical features of PBL, complex karyotype, and *c-MYC* rearrangement, which suggested that c-MYC dysregulation contributes to the plasmablastic morphology and aggressive clinical behavior in terminally differentiated B-cell neoplasms [124].

## 7. c-MYC in Mantle Cell Lymphoma

Mantle cell lymphoma (MCL) is a mature B-cell neoplasm with characteristic histological features and t(11;14)(q13;q32) juxtaposing *IGH* and *CCND1* gene loci [125]. The translocation results in deregulated expression of cyclin D1, which drives lymphoma cells through G1/S transition. The MCL cells are usually small mature lymphoid cells with mature chromatin and indistinct nuclei, but aggressive variants with lymphoblasts-like cells (blastoid variant) or DLBCL-like cells (pleomorphic variant) are also not uncommonly seen. While MCL is considered a progressively aggressive B-cell lymphoma, the clinical behavior is heterogeneous, ranging from indolent disease to highly aggressive blastoid and pleomorphic variants [126,127,128]. There is a correlation of cytogenetic changes with the clinical and morphologic features in MCL. The indolent MCL often shows simple karyotype, while the blastoid and pleomorphic MCLs most commonly have complex karyotype with highly unstable genomes and large numbers of secondary changes [129]. Frequent gains and losses in chromosomal loci involving genes important for genomic stability, proliferation, and apoptosis, such as *TP53*, *ATM*, c-*MYC*, *BMI1*, *CDK4*, and *BCL2*, are identified by comparative genomic hybridization microarray (CGH) [129,130]. c-MYC has been shown to cooperate with transcriptionally activated cyclin D1 in oncogenic transformation of B-cell lymphomas in transgenic mice [131]. BCR signaling promotes tumor proliferation and survival in MCL [132]. The activation of BCR causes MALT1 and BCL10 recruitment to CARD11, resulting in CARD11–BCL10–MALT1 (CBM) complex formation and subsequent activation of the nuclear factor kappa-B (NF-κB) [133,134]. MALT1 thus, is constitutively activated in a subset of MCL cases due to BCR activation and induces c-MYC expression by increasing c-MYC protein stability [135]. Accordingly, c-MYC expression is detected in approximately 73% of the cases, but the percentage of positive cells is frequently low (1–25% positive) [53].

MCL with 8q24/*c-MYC* abnormalities, referred by some as “double-hit” MCL, is rare [136,137,138,139,140,141,142,143,144,145]. Most cases have highly aggressive disease with a high-risk MCL international prognostic index, and high mortality within the first two years of diagnosis despite aggressive chemotherapy. Patients typically present with Ann Arbor stage III/IV disease with frequent involvement of bone marrow and peripheral blood in addition to the lymph node. *c-MYC* rearrangement occurs either initially at the time of diagnosis or is acquired during disease progression [144]. Histologically, the “double-hit” MCLs are nearly always of the blastoid variant, some with prominent nucleoli, and show high p53 expression and proliferation index by immunohistochemical stain with Ki-67 [136,144,145]. The lymphoma cells show strong expression of c-MYC in the majority of cells by immunohistochemistry [144]. They are more likely to show aberrant expression of CD10. The blastoid morphology correlates with acquisition of *c-MYC* rearrangement, as the MCL without *c-MYC* rearrangement display classic lymphocytic morphology [144]. Complex karyotype is invariably present in addition to t(11;14) (q13;q32). In “double-hit” MCL with *c-MYC* rearrangement, about half cases show translocation with the IGH or IG light chain gene loci, and the other half show non-IG translocation with *c-MYC*. The cooperation of cyclin D1 and c-MYC in generating blastoid MCL has been demonstrated in mouse models [146]. Similar to the atypical DHL, MCL with *c-MYC* amplification shows similar clinicopathologic features with “double-hit” MCL with *c-MYC* rearrangement [145]. Therefore, it appears that these cases should be similarly designated as atypical “double-hit” MCL, and belong to the same extreme spectrum of MCL as “double-hit” MCL, as they share the same underlying physiology of unregulated c-MYC expression. It should be noted that gains of 8q24/*c-MYC* frequently detected by CGH do not correlate with *c-MYC* amplification [147,148,149]. While FISH, PCR and high density SNP microarray are well suited in detecting *c-MYC* amplification, FISH is still the most common technique available clinically for both detecting *c-MYC* rearrangement and amplification.

## 8. c-MYC in Low Grade B-Cell Lymphomas

c-MYC protein expression identified by immunohistochemistry is commonly seen in low-grade B-cell lymphomas, including FL, chronic lymphocytic leukemia/small lymphocytic lymphoma (CLL/SLL), and splenic, extranodal, or nodal marginal zone lymphoma, but nearly always in a minority of lymphoma cells (<25%) [53]. The expression is highly variable and correlates with clinical behavior [53]. One study suggests that c-MYC overexpression predicts large cell transformation and is an independent, poor prognostic marker in extranodal marginal zone lymphoma of mucosa-associated lymphoid tissue (MALT lymphoma) at levels with ≥20% positive cells [150]. Craig et al. showed that c-MYC-mediated repression of microRNA-34a, which has a strong antiproliferative property via *FoxP1* targeting, likely promoted large cell transformation in MALT lymphoma [151]. Conversely, *c-MYC* rearrangement is rare in low-grade B-cell lymphomas, and is most commonly observed in FL and CLL/SLL. While *c-MYC* rearrangement is typically associated with an inferior prognosis and poor response to therapy in DLBCL, the clinical significance of *c-MYC* rearrangement in the low-grade B-cell lymphoma is less clear and should be interpreted in the context of overall cytogenetic changes.

CLL/SLL is the most common low-grade B-cell lymphoproliferative disorder in the Western world [58]. It most frequently involves peripheral blood and bone marrow, but involvement in the lymph node and spleen is also a common clinical presentation. A small subset of patients with CLL/SLL will undergo Richter’s transformation to DLBCL or rarely classical Hodgkin lymphoma. Histologically, CLL/SLL is composed of monotonous population of small mature lymphoid cells with round to mildly irregular nuclei with densely clumped chromatin and low proliferation rate, admixed with characteristic proliferation centers. The proliferation centers are composed of larger prolymphocytes and paraimmunoblasts and usually show moderate to high proliferation rate. The proliferation centers are believed to be the sites of B-cell receptor (BCR) signaling which promote CLL/SLL cell proliferation [152]. Gibson et al. showed that the cells in the proliferation centers of CLL/SLL were positive for c-MYC protein expression by immunohistochemistry, with the majority of cases demonstrating >25% c-MYC positive cells [153]. The c-MYC expression in the proliferation centers is not due to *c-MYC* rearrangement or amplification, which were not convincingly identified in this series [153]. As *c-MYC* is a major gene activated by BCR signaling pathway, the expression of c-MYC is postulated to be the result of surface IgM induced activation of MEK1/2-ERK1/2 within the proliferation centers [154,155]. The frequency of *c-MYC* rearrangement in CLL/SLL varies greatly in different studies. Nelson et al. showed no c-MYC rearrangement by FISH in 109 cases of CLL/SLL [156], and Li et al. identified 0.7% CLL/SLL with 8q24/MYC rearrangement in approximately 4500 cases [157]. In Li’s study, *c-MYC* is most frequently rearranged with immunoglobulin heavy chain gene locus in the t(8;14)(q24·1;q32), followed by light chain loci in t(8;22)(q24·1;q11·2), or t(2;8)(p12;q24·1). *c-MYC* rearrangement in CLL/SLL is often acquired during the course of disease and is associated with increased prolymphocytes or Richter’s transformation [157,158]. *c-MYC* rearrangement is frequently seen with concomitant adverse cytogenetic markers of CLL/SLL, such as del(11q) and/or del(17p)/monosomy 17, which helps explain the poor clinical outcomes in these patients [159]. However, the clinical significance of *c-MYC* rearrangement in CLL/SLL is likely dependent on the karyotype. If present in a non-complex karyotype, patients usually respond well to risk-adapted therapies and can achieve remission, while cases with a complex karyotype often show Richter’s transformation and an aggressive and refractory clinical course [157]. In a cohort of 156 patients with B-cell neoplasms harboring c*-MYC* rearrangement 33 patients (21%) carried the diagnosis of CLL. Haberl et al. showed single hit *c-MYC* rearrangement in 88% of CLL/SLL cases and multi-hit *c-MYC* rearrangement (with *BCL2*, or *BCL6*) in 12% of CLL/SLL cases [160]. In this study, CLL/SLL with *c-MYC* rearrangement often showed a non-complex karyotype (85%), non-immunoglobulin loci as the most frequent *c-MYC* partner genes (61%), high frequency of *SF3B1* mutations, and absence of *ID3* mutations. Additionally, when compared to CLL cases without *c-MYC* translocation, CLL with *c-MYC* translocations showed three-fold increase in 17p deletions. (24% vs. 7%) [160].

FL is one of the most common low-grade B-cell lymphomas in the Western world [58]. It is composed of neoplastic germinal center B-cells (centrocytes and centroblasts) in a follicular, follicular/diffuse, or less commonly diffuse pattern. FL is graded based on the number of centroblasts in the follicles, with grade 1–2 FL showing ≤15 centroblasts/high power field and grade 3 FL having >15 centroblasts/high power field. The hallmark cytogenetic aberration of FL is the translocation/fusion of *BCL2* and *IgH* leading to t(14;18)(q32;q21). This translocation leads to overexpression of BCL2 and suppression of apoptosis and survival of neoplastic germinal center B cells. As *BCL2* rearrangement is the one of the two “hits” in double-hit lymphoma, acquisition of *c-MYC* rearrangement during disease progression leads to histologic transformation to high-grade B-cell lymphoma/DHL and rarely plasmablastic lymphoma or blastoid transformation of FL/B-lymphoblastic lymphoma [101,161,162]. As expected, the *c-MYC* rearrangement in these cases usually occurs in the setting of a complex karyotype. Therefore, DHL defined cytogenetically by *c-MYC* and *BCL2* rearrangements comprise both de novo and secondary lymphomas transformed from FL. Approximately 25–35% of FL eventually transforms to DLBCL, and most transformed FL are not DHL. There is a low frequency of *c-MYC* rearrangement, amplification or somatic mutations associated with the transformation of FL to DLBCL [163,164,165]. Therefore, *c-MYC* genetic abnormalities are not the primary events driving transformation of FL to non-DHL DLBCL. Transformation of FL proceeds by two predominant pathways, one characterized by higher proliferation rate and the other without increased proliferation [166]. The expression of c-MYC and downstream target genes are secondary events and are part of the proliferation signature. Recurrent changes in genes controlling cell cycle progression and DNA damage responses (*CDKN2A/B* and *TP53*) with subsequent genomic instability and dysregulated proliferation are important in the transformation of FL [166].

Non-transformed FL with concurrent c-*MYC* and *BCL2* rearrangements, so-called “double-hit” FL, occur rarely and are only reported as single case reports or as small case series in the literature [163,167,168,169,170,171,172]. The majority of the reported cases were low-grade FLs. While some patients had more aggressive clinical course and died within two years of diagnosis [163,169,170], there were also cases with a more indolent clinical course as typically seen in low-grade FL [168]. Miao et al. recently reported seven cases of “double-hit” FL with most showing low-grade histology with only focal high-grade FL or focally high proliferation rate [172]. In this series, most patients had advance stage disease. Patients treated with intense chemotherapy for DHL etoposide phosphate, prednisone, oncovin, cyclophosphamide doxorubicin hydrochloride and rituximab (EPOCH-R) responded well and attained complete remission. It is difficult to determine if the clinical course and prognosis correlate with cytogenetic findings and proliferation rate of lymphoma cells, due to the paucity of reported cases. The three cases reported by Christie et al. all had complex karyotypes, but showed different clinical courses with two indolent cases and one with high-grade transformation of FL with subsequent fatality [168]. Because pathologic and genetic features are not defined in predicting prognosis, patients with “double-hit” FL may benefit from more intense chemotherapy regimens reserved for DHL after careful clinicopathologic evaluation on an individual basis. c-MYC expression by immunohistochemistry is observed in approximately 1–25% of the cells in 60–70% of FL, and rarely in >25% of the cells [53]. Clearly c-MYC expression is not related to *c-MYC* rearrangement and does not predict prognosis in FL.

## 9. Conclusions

As a global transcription factor, c-MYC transforms cells by unregulated expression rather than oncogenic mutations. In lymphomas, *c-MYC* genetic abnormalities are restricted almost exclusively to B-cell lymphomas, and include primarily rearrangements and amplifications. *c-MYC* rearrangement is the most extensively studied, and B-cell lymphomas with *c-MYC* rearrangements show a spectrum of clinical course ranging from indolent low-grade B-cell lymphoma to the highly aggressive DHL. The clinical significance of *c-MYC* rearrangement in B-cell lymphomas correlates with overall genetic context of the lymphoma. In general, *c-MYC* rearranged B-cell lymphomas with a simple karyotype such as that usually seen in BL respond better to chemotherapy, while those with a complex karyotype, as is typically seen in DHLs, show a very poor clinical course and are refractory to conventional chemotherapy and even hematopoietic stem cell transplant. Clinically, it is important to correctly diagnose DHLs as these patients may benefit from clinical trials with novel targeted agents or optimization of standard immunochemotherapy regimens.
